# A super-spreading ewe infects hundreds with Q fever at a farmers' market in Germany

**DOI:** 10.1186/1471-2334-6-147

**Published:** 2006-10-06

**Authors:** Klaudia Porten, Jürgen Rissland, Almira Tigges, Susanne Broll, Wilfried Hopp, Mechthild Lunemann, Ulrich van Treeck, Peter Kimmig, Stefan O Brockmann, Christiane Wagner-Wiening, Wiebke Hellenbrand, Udo Buchholz

**Affiliations:** 1 Robert Koch-Institute: Seestrasse 10, 13353 Berlin, Germany; 2 Soest County Health Department: Hoher Weg 1–3, 59494 Soest, Germany; 3 Soest Veterinary Health Department: Hoher Weg 1–3, 59494 Soest, Germany; 4 Institute of Public Health, State of Northrhine Westphalia: LÖGD NRW, Standort Münster, von-Stauffenbergstraße 36, 48151 Münster, Germany; 5 National Consulting Laboratory for *Coxiella burnetii *at the Baden-Württemberg State Health Office: Konsiliarlaboratorium für Coxiella burnetii, Landesgesundheitsamt, Regierungspräsidium Stuttgart Wiederholdstraße 15, 70174 Stuttgart, Germany

## Abstract

**Background:**

In May 2003 the Soest County Health Department was informed of an unusually large number of patients hospitalized with atypical pneumonia.

**Methods:**

In exploratory interviews patients mentioned having visited a farmers' market where a sheep had lambed. Serologic testing confirmed the diagnosis of Q fever. We asked local health departments in Germany to identiy notified Q fever patients who had visited the farmers market. To investigate risk factors for infection we conducted a case control study (cases were Q fever patients, controls were randomly selected Soest citizens) and a cohort study among vendors at the market. The sheep exhibited at the market, the herd from which it originated as well as sheep from herds held in the vicinity of Soest were tested for *Coxiella burnetii *(*C. burnetii*).

**Results:**

A total of 299 reported Q fever cases was linked to this outbreak. The mean incubation period was 21 days, with an interquartile range of 16–24 days. The case control study identified close proximity to and stopping for at least a few seconds at the sheep's pen as significant risk factors. Vendors within approximately 6 meters of the sheep's pen were at increased risk for disease compared to those located farther away. Wind played no significant role. The clinical attack rate of adults and children was estimated as 20% and 3%, respectively, 25% of cases were hospitalized. The ewe that had lambed as well as 25% of its herd tested positive for *C. burnetii *antibodies.

**Conclusion:**

Due to its size and point source nature this outbreak permitted assessment of fundamental, but seldom studied epidemiological parameters. As a consequence of this outbreak, it was recommended that pregnant sheep not be displayed in public during the 3^rd ^trimester and to test animals in petting zoos regularly for *C. burnetii*.

## Background

Q fever is a worldwide zoonosis caused by *Coxiella burnetii *(*C. burnetii*), a small, gram-negative obligate intracellular bacterium. *C. burnetii *displays antigenic variation with an infectious phase I and less infectious phase II. The primary reservoir from which human infection occurs consists of sheep, goat and cattle. Although *C. burnetii *infections in animals are usually asymptomatic, they may cause abortions in sheep and goats[1]. High concentrations of *C. burnetii *can be found in birth products of infected mammals[2]. Humans frequently acquire infection through inhalation of contaminated aerosols from parturient fluids, placenta or wool[1]. Because the infectious dose is very low[3] and *C. burnetii *is able to survive in a spore-like state for months to years, outbreaks among humans have also occurred through contaminated dust carried by wind over large distances [4-6].

*C. burnetii *infection in humans is asymptomatic in approximately 50% of cases. Approximately 5% of cases are hospitalized, and fatal cases are rare[1]. The clinical presentation of acute Q fever is variable and can resemble many other infectious diseases[2]. However, the most frequent clinical manifestation of acute Q fever is a self-limited febrile illness associated with severe headache. Atypical pneumonia and hepatitis are the major clinical manifestations of more severe disease. Acute Q fever may be complicated by meningoencephalitis or myocarditis. Rarely a chronic form of Q fever develops months after the acute illness, most commonly in the form of endocarditis[1]. Children develop clinical disease less frequently[7,8]. Because of its non-specific presentation Q fever can only be suspected on clinical grounds and requires serologic confirmation. While the indirect immunofluorescence assay (IFA) is considered to be the reference method, complement fixation (CF), ELISA and microagglutination (MA) can also be used[9]. Acute infections are diagnosed by elevated IgG and/or IgM anti-phase II antibodies, while raised anti-phase I IgG antibodies are characteristic for chronic infections[1].

In Germany, acute Q fever is a notifiable disease. Between 1991 and 2000 the annual number of cases varied from 46 to 273 cases per year [10]. In 2001 and 2002, 293 and 191 cases were notified, respectively[11,12].

On May 26, 2003 the health department of Soest was informed by a local hospital of an unusually large number of patients with atypical pneumonia. Some patients reported having visited a farmers' market that took place on May 3 and 4, 2003 in a spa town near Soest. Since the etiology was unclear, pathogens such as SARS coronavirus were considered and strict infection control measures implemented until the diagnosis of Q fever was confirmed.

An outbreak investigation team was formed and included public health professionals from the local health department, the local veterinary health department, the state health department, the National Consulting Laboratory (NCL) for Coxiellae and the Robert Koch-Institute (RKI), the federal public health institute. Because of the size and point source appearance of the outbreak the objective of the investigation was to identify etiologic factors relevant to the prevention and control of Q fever as well as to assess epidemiological parameters that can be rarely studied otherwise.

## Methods

### Hypothesis generation

On May 26 and 27, 2003 we conducted exploratory interviews with patients in Soest hospitalized due to atypical pneumonia.

### Etiologic confirmation

Attending physicians were requested to test serum of patients with atypical pneumonia for *Mycoplasma pneumoniae*, *Chlamydia pneumoniae*, *Legionella pneumophila*, *Coxiella burnetii*, Influenza A and B, Parainfluenza 1–3, Adenovirus and Enterovirus. Throat swabs were tested for Influenza virus, Adenovirus and *SARS-Coronavirus*. Laboratory confirmation of an acute Q fever infection was defined as the presence of IgM antibodies against phase II *C. burnetii *antigens (ELISA or IFA), a 4-fold increase in anti-phase II IgG antibody titer (ELISA or IFA) or in anti phase II antibody titer by CF between acute and convalescent sera. A chronic infection was confirmed when both anti-phase I IgG and anti-phase II IgG antibody titers were raised.

### Investigation of exposed patients with valvular heart defects and pregnant women

Because patients with valvular heart defects and pregnant women are at high risk of developing chronic infection[13,14] we alerted internists and gynaecologists through the journal of the German Medical Association and asked them to send serum samples to the NCL if they identified patients from these risk groups who had been at the farmers' market during the outbreak.

### First case control study (CCS1)

The objective of the first case control study was to establish whether there was a link between the farmers' market and the outbreak and to identify other potential risk factors. We conducted telephone interviews using a standardised questionnaire that asked about attendance at the farmers' market, having been within 1 km distance of one of 6 sheep flocks in the area, tick bites and consumption of unpasteurized milk, sheep or goat cheese. For the purpose of CCS1 we defined a case (CCS1 case) as an adult resident of the town of Soest notified to the statutory surveillance system with Q fever, having symptom onset between May 4 and June 3, 2003. Exclusion criterion was a negative IgM-titer against phase II antigens. Two controls per case were recruited from Soest inhabitants by random digit dialing.

We calculated the attributable fraction of cases exposed to the farmers' market on May 4 (AFE) as (OR-1)/OR and the attributable fraction for all cases due to this exposure as:

Attributable fraction=AFE*Number of cases exposedAll cases
 MathType@MTEF@5@5@+=feaafiart1ev1aaatCvAUfKttLearuWrP9MDH5MBPbIqV92AaeXatLxBI9gBaebbnrfifHhDYfgasaacH8akY=wiFfYdH8Gipec8Eeeu0xXdbba9frFj0=OqFfea0dXdd9vqai=hGuQ8kuc9pgc9s8qqaq=dirpe0xb9q8qiLsFr0=vr0=vr0dc8meaabaqaciaacaGaaeqabaqabeGadaaakeaacqqGbbqqcqqG0baDcqqG0baDcqqGYbGCcqqGPbqAcqqGIbGycqqG1bqDcqqG0baDcqqGHbqycqqGIbGycqqGSbaBcqqGLbqzcqqGGaaicqqGMbGzcqqGYbGCcqqGHbqycqqGJbWycqqG0baDcqqGPbqAcqqGVbWBcqqGUbGBcqGH9aqpcqqGbbqqcqqGgbGrcqqGfbqrcqGGQaGkdaWcaaqaaiabb6eaojabbwha1jabb2gaTjabbkgaIjabbwgaLjabbkhaYjabbccaGiabb+gaVjabbAgaMjabbccaGiabbogaJjabbggaHjabbohaZjabbwgaLjabbohaZjabbccaGiabbwgaLjabbIha4jabbchaWjabb+gaVjabbohaZjabbwgaLjabbsgaKbqaaiabbgeabjabbYgaSjabbYgaSjabbccaGiabbogaJjabbggaHjabbohaZjabbwgaLjabbohaZbaaaaa@764A@

### Determination of outbreak size and descriptive epidemiology

The farmers' market was held in a spa town near Soest with many visitors from other areas of the state and even the entire country. To determine the outbreak size we therefore asked local public health departments in Germany to ascertain a possible link to the farmers' market in Soest for all patients notified with Q-fever. A case in this context ("notified case") was defined as any person with a clinical diagnosis compatible with Q fever with or without laboratory confirmation and history of exposure to the farmers' market.

Local health departments also reported whether a notified case was hospitalized. To obtain an independent, second estimate of the proportion of hospitalizations among symptomatic patients beyond that reported through the statutory surveillance system we calculated the proportion of hospitalized patients among those persons fulfilling the clinical case definition (as used in the vendors' study (s.b.)) identified through random sampling of the Soest population (within CCS2 (s.b.)) as well as in two cohorts (vendors' study and the 9 sailor friends (see below)).

### Second case control study (CCS2)

The objective of CCS2 was to identify risk factors associated with attendance of the farmers' market on the second day. We used the same case definition as in CCS1, but included only persons that had visited the farmers' market on May 4, the second day of the market. We selected controls again randomly from the telephone registry of Soest and included only those persons who had visited the farmers' market on May 4 and had not been ill with fever afterwards. Potential controls who became ill were excluded for analysis in CCS2, but were still fully interviewed. This permitted calculation of the attack rate among visitors to the market (see below "Estimation of the overall attack rate") and gave an estimate of the proportion of clinically ill cases that were hospitalized (s.a.).

### Cohort study on vendors

In the vendors' study we investigated whether the distance of the vendor stands from the sheep pen or dispersion of *C. burnetii *by wind were relevant risk factors for acquiring Q fever. We obtained a list of all vendors including the approximate location of the stands from the organizer. In addition we asked the local weather station for the predominant wind direction on May 4, 2003. Telephone interviews were performed using standardized questionnaires. A case was defined as a person with onset of fever between May 4 and June 3, 2003 and at least three of the following symptoms: headache, cough, dyspnea, joint pain, muscle pain, weight loss of more than 2 kg, fatigue, nausea or vomiting.

**Figure 1:**
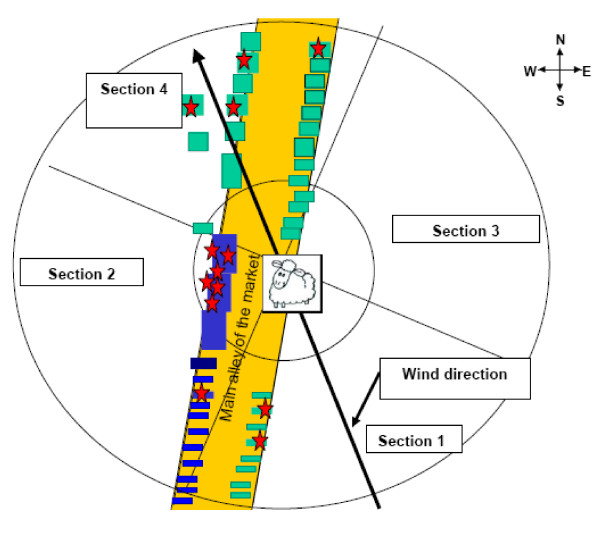
Schematic display of the farmers' market on May 3 and 4, 2003 and the location of the stands of the vendors. On May 4, the predominant direction of the wind was from south-south-east. Red stars represent cases among vendors.

The relative distance of the stands to the sheep pen was estimated by counting the stands between the sheep pen and the stand in question. Each stand was considered to be one stand unit (approximately 3 meters). Larger stands were counted as 2 units. The direction of the wind in relation to the sheep pen was defined by dividing the wind rose (360°) in 4 equal parts of 90°. The predominant wind direction during the market was south-south-east (Figure 1). For the purpose of the analysis we divided the market area into 4 sections with the sheep pen at its center. In section 1 the wind was blowing towards the sheep pen (plus minus 45°). Section 4 was on the opposite side, i.e. where the wind blew from the sheep pen towards the stands, and sections 2 and 3 were east and west with respect to the wind direction, respectively. Location of the stands in reference to the sheep pen was thus defined in two ways: as the absolute distance to the sheep pen (in stand units or meters) and in reference to the wind direction.

### Estimation of the proportion of symptomatically ill persons among those serologically confirmed and calculation of the overall attack rate among adults and children

We identified a small cohort of 9 sailor friends who visited the farmers' market on May 4, 2003. All of these were serologically tested independently of symptoms. We could therefore calculate the **proportion of laboratory confirmed persons **who met the clinical case definition (as defined in the cohort study on vendors).

The overall **attack rate among adults was estimated based on the following sources**:

(1) Interviews undertaken for recruitment of controls for CCS2 allowed the proportion of adults that acquired symptomatic Q fever among those who visited the farmers' market on the second day;

(2) Interviews of cases and controls in CCS2 yielded information about accompanying adults and how many of these became later "ill with fever";

(3) Results of the small cohort of 9 sailor friends (s.a.);

(4) Results from the cohort study on vendors.

Local health departments that identified outbreak cases of Q fever (s.a. "determination of outbreak size and descriptive epidemiology") interviewed patients about the number of persons that had accompanied them to the farmers' market and whether any of these had become ill with fever afterwards. However, as there was no differentiation between adults and children, calculations to estimate the attack rate among adults were performed both with and without this source.

To count cases in (1), (3) and (4) we used the clinical case definition as defined in the cohort study on vendors.

For the calculation of the **attack rate among children **(less than 18 years old) we first estimated the number of children who visited the market (denominator). To do this we assumed that the proportion of children (= 1 - proportion of adults) visiting the farmers' market on May 4 as elicited in CCS2 was the same for all visitors. The number of children that visited the market could then be estimated from the total number of visitors as estimated by the organizers. We then estimated the number of symptomatic children (numerator). For this we assumed that the proportion of children with Q fever that were seen by physicians and were consequently notified was the same as that of adults. It was calculated as:

Porportion reported=number of notified adultsnumber of visiting adults*attack rate among adults
 MathType@MTEF@5@5@+=feaafiart1ev1aaatCvAUfKttLearuWrP9MDH5MBPbIqV92AaeXatLxBI9gBaebbnrfifHhDYfgasaacH8akY=wiFfYdH8Gipec8Eeeu0xXdbba9frFj0=OqFfea0dXdd9vqai=hGuQ8kuc9pgc9s8qqaq=dirpe0xb9q8qiLsFr0=vr0=vr0dc8meaabaqaciaacaGaaeqabaqabeGadaaakeaacqqGqbaucqqGVbWBcqqGYbGCcqqGWbaCcqqGVbWBcqqGYbGCcqqG0baDcqqGPbqAcqqGVbWBcqqGUbGBcqqGGaaicqqGYbGCcqqGLbqzcqqGWbaCcqqGVbWBcqqGYbGCcqqG0baDcqqGLbqzcqqGKbazcqGH9aqpdaWcaaqaaiabb6gaUjabbwha1jabb2gaTjabbkgaIjabbwgaLjabbkhaYjabbccaGiabb+gaVjabbAgaMjabbccaGiabb6gaUjabb+gaVjabbsha0jabbMgaPjabbAgaMjabbMgaPjabbwgaLjabbsgaKjabbccaGiabbggaHjabbsgaKjabbwha1jabbYgaSjabbsha0jabbohaZbqaaiabb6gaUjabbwha1jabb2gaTjabbkgaIjabbwgaLjabbkhaYjabbccaGiabb+gaVjabbAgaMjabbccaGiabbAha2jabbMgaPjabbohaZjabbMgaPjabbsha0jabbMgaPjabb6gaUjabbEgaNjabbccaGiabbggaHjabbsgaKjabbwha1jabbYgaSjabbsha0jabbohaZjabcQcaQiabbggaHjabbsha0jabbsha0jabbggaHjabbogaJjabbUgaRjabbccaGiabbkhaYjabbggaHjabbsha0jabbwgaLjabbccaGiabbggaHjabb2gaTjabb+gaVjabb6gaUjabbEgaNjabbccaGiabbggaHjabbsgaKjabbwha1jabbYgaSjabbsha0jabbohaZbaaaaa@A81B@

Thus the true number of children with Q fever was estimated by the number of reported children divided by the estimated proportion reported. Then the attack rate among children could be estimated as follows:

Attack rate among children=estimated true number of children with Q feverestimated number of children at the market
 MathType@MTEF@5@5@+=feaafiart1ev1aaatCvAUfKttLearuWrP9MDH5MBPbIqV92AaeXatLxBI9gBaebbnrfifHhDYfgasaacH8akY=wiFfYdH8Gipec8Eeeu0xXdbba9frFj0=OqFfea0dXdd9vqai=hGuQ8kuc9pgc9s8qqaq=dirpe0xb9q8qiLsFr0=vr0=vr0dc8meaabaqaciaacaGaaeqabaqabeGadaaakeaacqqGbbqqcqqG0baDcqqG0baDcqqGHbqycqqGJbWycqqGRbWAcqqGGaaicqqGYbGCcqqGHbqycqqG0baDcqqGLbqzcqqGGaaicqqGHbqycqqGTbqBcqqGVbWBcqqGUbGBcqqGNbWzcqqGGaaicqqGJbWycqqGObaAcqqGPbqAcqqGSbaBcqqGKbazcqqGYbGCcqqGLbqzcqqGUbGBcqGH9aqpdaWcaaqaaiabbwgaLjabbohaZjabbsha0jabbMgaPjabb2gaTjabbggaHjabbsha0jabbwgaLjabbsgaKjabbccaGiabbsha0jabbkhaYjabbwha1jabbwgaLjabbccaGiabb6gaUjabbwha1jabb2gaTjabbkgaIjabbwgaLjabbkhaYjabbccaGiabb+gaVjabbAgaMjabbccaGiabbogaJjabbIgaOjabbMgaPjabbYgaSjabbsgaKjabbkhaYjabbwgaLjabb6gaUjabbccaGiabbEha3jabbMgaPjabbsha0jabbIgaOjabbccaGiabbgfarjabbccaGiabbAgaMjabbwgaLjabbAha2jabbwgaLjabbkhaYbqaaiabbwgaLjabbohaZjabbsha0jabbMgaPjabb2gaTjabbggaHjabbsha0jabbwgaLjabbsgaKjabbccaGiabb6gaUjabbwha1jabb2gaTjabbkgaIjabbwgaLjabbkhaYjabbccaGiabb+gaVjabbAgaMjabbccaGiabbogaJjabbIgaOjabbMgaPjabbYgaSjabbsgaKjabbkhaYjabbwgaLjabb6gaUjabbccaGiabbggaHjabbsha0jabbccaGiabbsha0jabbIgaOjabbwgaLjabbccaGiabb2gaTjabbggaHjabbkhaYjabbUgaRjabbwgaLjabbsha0baaaaa@BF38@

Because this calculation was based on several assumptions (number of visitors, proportion of adult visitors and clinical attack rate among adults) we performed a sensitivity analysis where the values of these variables varied.

### Environmental investigation

Serum was collected from all sheep and cows displayed in the farmers' market as well as from all sheep of the respective home flocks (70 animals). Samples of 25 sheep from five other flocks in the Soest area were also tested for *C. burnetii*. Tests were performed by ELISA with a phase I and phase II antigen mixture.

### Statistical analysis

We conducted statistical analysis with Epi Info, version 6.04 (CDC, Atlanta, USA). Dichotomous variables in the case control and cohort studies were compared using the Chi-Square test and numerical variables using the Kruskal-Wallis test. P-values smaller than 0.05 were considered statistically significant.

### Ethical issues

The outbreak investigation was conducted within the framework of the Communicable Diseases Law Reform Act of Germany. Mandatory regulations were observed.

## Results

### Hypothesis generation

Patients at the local hospital in Soest reported that a farmers' market had taken place on May 3 and 4, 2003 in a spa town close to the town of Soest. It was located in a park along the main promenade, spanning a distance of approximately 500 meters. The market attracted mainly three groups of people: locals, inhabitants of the greater Soest region, patients from the spa sanatoria and their visiting family or friends. Initial interviewees mentioned also that they had spent time at the sheep pen watching newborn lambs that had been born in the early morning hours of May 4, 2003. The ewe had eaten the placenta but the parturient fluid on the ground had merely been covered with fresh straw.

### Etiologic confirmation

Overall 171 (65%) of 263 serum samples submitted to the NCL were positive for IgM anti-phase II antibodies by ELISA. Results of throat swabs and serum were negative for other infectious agents.

### First case control study

For CCS1 we included the first 22 cases notified to the statutory surveillance system who were willing to participate in the study, as well as 45 controls. All CCS1 cases reported that they had fever (100%), 20/21 (95%) reported fatigue, 19/20 (95%) had weight loss of more than 2 kg, 20/22 (91%) had joint pain, 14/19 (74%) had muscle pain, and 13/22 (59%) had headache.

Analysis showed that CCS1 cases were 210 times more likely to have visited the farmers' market on May 4, 2003, (odds ratio (OR) = 210; 95% confidence interval (CI) = 22 – 3601; p < 0.001), while there was no significant association with a visit to the market on May 3, 2003, (OR = 0.0; 95%CI = 0.0–4.8; p = 0.55). Cases were not more likely than controls to be male than female, to have been within 1 km of a grazing sheep flock in the area, to have been bitten by a tick, to have consumed raw milk, goat or sheep cheese. Two CCS1 cases did not visit the farmers' market on May 4, but on May 5 they were in the park where the market had taken place.

AFE of exposed cases for visiting the market on May 4 was 99.5% ((210-1)/210) and the attributable fraction among cases for this exposure was 90.5% (99.5% * [20/22]).

### Case finding and descriptive epidemiology

**Figure 2:**
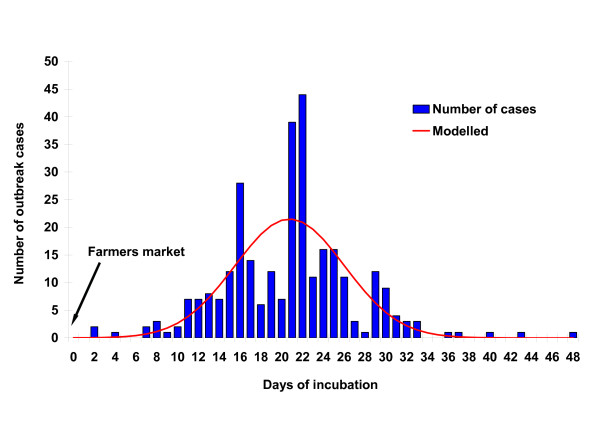
Outbreak cases of Q fever linked to the farmers' market on May 4, 2003 as notified by local health departments (N = 299). The interval between the day of onset and May 4 yields the incubation period. Superimposed is a modeled normal distribution of all outbreak cases.

**Figure 3:**
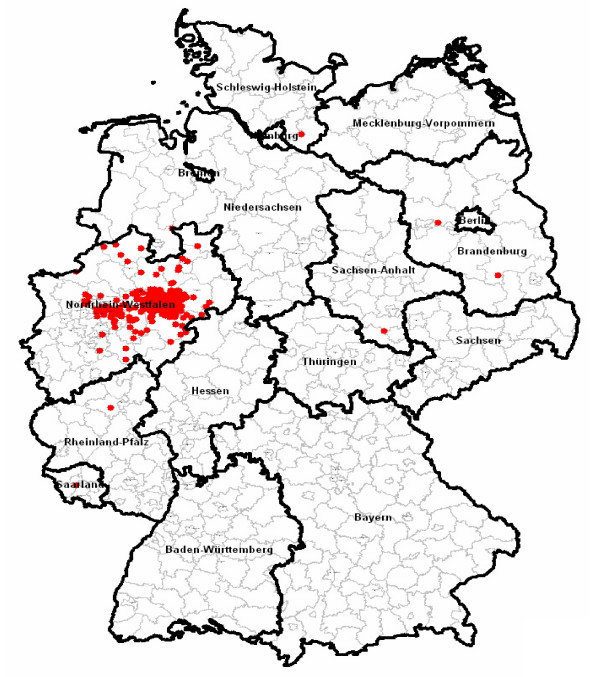
Geographical location of Q fever outbreak cases notified to the statutory surveillance system.

**Figure 4:**
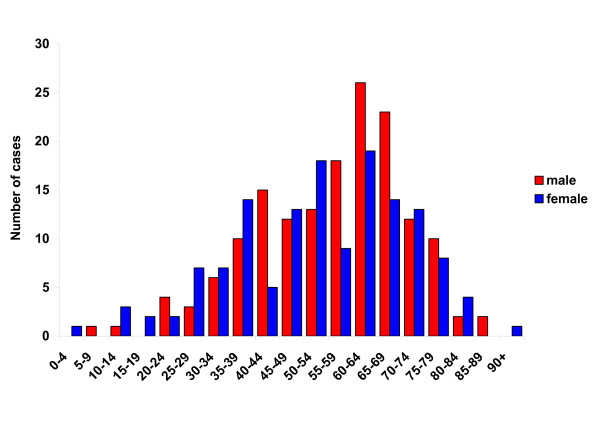
Age and sex distribution of notified cases of the Q fever outbreak associated with the farmers' market in a spa town near Soest; 2003.

299 cases of Q fever linked to the farmers' market were notified to the statutory surveillance system. The date of illness onset ranged between May 6 and June 21, 2003 (median, May 25; mode, May 26 (44 notified cases)). Taking May 4, 2003 as the day of exposure for outbreak cases, the incubation period ranged from 2 to 48 days, with a median of 21 days and an interquartile range (comprising 50% of notified cases) of 14 to 24 days (Figure 2). If we assume that symptom onset in cases was normally distributed with a mean of 21 days, 95% of cases (mean +/- 2 standard deviations) had their onset between day 10 and 31. The two notified cases with early onset on May 6 and 8, respectively, were laboratory confirmed and additional interviews did not reveal any additional risk factors. Of the 298 cases with known gender, 158 (53%) were male and 140 (47%) were female. Of the notified cases, 189 (63%) were from the county of Soest, 104 (35%) were from other counties in the same federal state (Northrhine Westphalia) and 6 (2%) were from five other federal states in Germany (Figure 3). Only eight (3%) cases were less than 18 years of age, the mean and median age was 54 and 56 years, respectively (Figure 4). 75 (25%) of 297 notified cases were hospitalized, none died. Calculation of the proportion of cases hospitalized through other information sources revealed that 4 of 19 (21%; 95% CI = 6–46%; (1/5 (CCS2), 2/11 (vendors study) and 1/3 (sailor friends)) clinically ill cases were hospitalized.

Laboratory confirmation was reported in 167 (56%) outbreak cases; 66 (22%) were confirmed by an increase in anti-phase II antibody titer (CF), 89 (30%) had IgM antibodies against phase II antigens, 11 (4%) were positive in both tests and one was confirmed by culture. No information was available as to whether the 132 (44%) cases without laboratory confirmation were laboratory tested.

### Investigation of exposed patients with valvular heart defects and pregnant women

18 patients with valvular heart defects and eleven pregnant women were examined. None of them had clinical signs of Q fever. Two (11%) of 18 cardiological patients and four (36%) of 11 pregnant women had an acute Q fever infection. During childbirth strict hygienic measures were implemented. Lochia and colostrum of all infected women were tested by polymerase chain reaction and were positive in only one woman (case 3; Table 1). Serological follow-up of the mothers detected chronic infection in the same woman (case 3) 12 weeks after delivery. One year follow-up of two newborn children (of cases 1 and 3) identified neither acute nor chronic Q fever infections.

**Table 1:** Epidemiological details of four pregnant women with acute Q fever infection

**Case number**	**Age**	**Week of exposure**	**Antibiotic treatment**	**Week of delivery**	**Delivery**	**Lochia and colostrum**	**Serological follow-up after delivery**
1	35	27	Erythromycin/Clarithromycin (3 weeks)	35	Primary caesarean	Negative	Negative
2	29	23	Trimethoprim-Sulfamethoxazol (1 week)	40	Primary caesarean	Negative	Negative
3	29	16	Trimethoprim-Sulfamethoxazol (1 week)	40	Primary caesarean	Positive	Chronic infection
4	25	9	Clarithromycin (until delivery)	40	Secondary caesarean	Negative	Negative

### Second case control study

We recruited 20 cases and 36 controls who visited the farmers' market on May 4 for the second case control study. They did not differ significantly in age and gender (OR for male sex = 1.7; 95%CI = 0.5–5.3; p = 0.26; p-value for age = 0.23). Seventeen (85%) of 20 cases indicated that they had seen the cow (that also was on display at the market next to the sheep) compared to 7 (32%) of 22 controls (OR = 12.1; 95%CI = 2.2–77.7; p =< 0.01). All cases, but only 19 (79%) of 24 controls remembered having seen the sheep (OR undefined; lower 95%CI = 1.1; p = 0.04). Fourteen (70%) of 20 CCS2 cases spent time within or directly at the gate of the sheep pen compared to 8 (32%) of 25 controls (OR = 5.0; 95%CI = 1.2–22.3; p = 0.03). Touching the sheep was also significantly more common among cases (5/20 (25%) CCS2 cases vs. 0/22 (0%) controls; OR undefined; lower 95% CI = 1.1; p = 0.02). 17 (85%) of 20 CCS2 cases, but only 6 (25%) of 24 controls stopped for at least a few seconds at or in the sheep pen, the reference for this variable was "having passed by the pen without stopping" (OR = 17.0; 95%CI = 3.0–112.5; p < 0.01). Among CCS2 cases, self-reported proximity to or time spent with/close to the sheep was not associated with a shorter incubation period.

### Cohort study on vendors

We were able to contact and interview 75 (86%) of 87 vendors, and received second hand information about 7 more (overall response rate: 94%). Fourty-five (56%) were male and 35 (44%) were female. 13 (16%) met the clinical case definition. Of the 11 vendors who worked within two stand units of the sheep pen, 6 (55%) became cases compared to only 7 (10%) of 70 persons who worked in a stand at a greater distance (relative risk (RR) = 5.5 (95%CI = 2.3–13.2; p = 0.002); Figure 1). Of these 7 vendors, 4 had spent time within 5 meters of the pen on May 4, one had been near the pen, but at a distance of more than 5 meters, and no information on this variable was available for the remaining 2. In the section of the market facing the wind coming from the pen (section 4, Figure 1), 4 (9%) of 44 vendors became cases, compared to 2 (13%) of 15 persons who worked in section 1 (p = 0.6). Among 22 persons who worked in stands that were perpendicular to the wind direction, 7 (32%) became cases.

### Estimation of the proportion of diseased among serologically confirmed and calculation of the overall attack rate among adults and children

In the small cohort of 9 sailors who visited the farmers' market on May 4, 2003, 6 had positive titers for *C. burnetii*, and 3 of these met the clinical case definition. Thus, in this small group 50% of persons with positive serology had manifest disease.

Pooled data on the **clinical attack rate among adults **who had visited the farmers' market on May 4, yielded an estimate of 20% (95%CI = 15–27%; Table 2). Inclusion of information on persons accompanying notified cases (fifth source) increased this estimate to 24% (58/242; 95%CI = 19–30%).

**Table 2:** Data sources used to calculate the clinical attack rate among adults; CCS2 = second case control study.

**Data source**	**III**	**Exposed**	**Attack rate**
(1) CCS2, interviewees in the telephone survey for the recruitment of controls	8	35	23%
(2) Adults who accompanied cases or controls (CCS2)	8	32	25%
(3) Cohort of sailor friends	3	9	33%
(4) Cohort of vendors	13	82	16%
Pooled (sources (1)-(4))	32	158	20%
(5) Persons accompanying notified cases	26	84	31%
Pooled (sources (1)-(5))	58	242	24%

Persons accompanying notified cases (source 5) were a mixture of adults and children and are therefore listed separately.

#### Clinical attack rate among children

**Figure 5:**
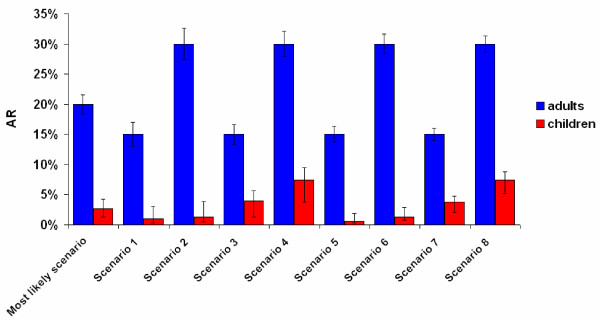
Attack rates among adults and children in a most likely scenario and 8 other scenarios. Most likely scenario: 3000 visitors, 83% adult visitors and 20% clinical attack rate among adults. Scenarios 1–8 varied in the assumptions made for "number of visitors", "proportion of adult visitors" and "attack rate among adults" (see Table 3). Displayed are attack rates and 95% confidence intervals.

First we calculated a **"most likely scenario" **before we varied the different assumptions in the calculation. The organizers estimated that on May 4 about 3000 persons visited the market. Based on data from CCS2 83% of visitors were adults and 17% were children. Thus the total number of adult visitors was estimated as 2490. With an attack rate among adults of 20% the estimated number of ill adults was 498. Since the number of notified adults was 291 the proportion of illl adults that was notified was 58%. If there were 2490 adult visitors then children comprised 510 (= 3000–2490) visitors. Assuming that the reporting rate among children is the same as in adults then the number of reported children/reporting rate gives the estimated number of ill children. This yields 8/0,58 = 14 ill children. Therefore the estimated AR among children was 14/510 = 2.7%. The total estimated number of persons with clinical Q fever would be 498 + 14 = 512. In the **sensitivity analysis **we varied the number of visitors between 2000 and 5000, the proportion of adult visitors between 60% and 90% and the attack rate among adults between 15% and 30% (Table 3). In all scenarios the AR among adults was significantly higher than that among children (Figure 5).

**Table 3:** Sensitivity analysis of estimated attack rates (AR) among children.

	**Most likely scenario**	**Scenario 1**	**Scenario 2**	**Scenario 3**	**Scenario 4**	**Scenario 5**	**Scenario 6**	**Scenario 7**	**Scenario 8**
Total number of visitors	3000	2000	2000	2000	2000	5000	5000	5000	5000
% adult visitors	83%	60%	60%	90%	90%	60%	60%	90%	90%
Number of adult visitors at farmers market	2490	1200	1200	1800	1800	3000	3000	4500	4500
Estimated AR (adults)	20%	15%	30%	15%	30%	15%	30%	15%	30%
Estimated number of ill adults	498	180	360	270	540	450	900	675	1350
Number of reported adults	291	291	291	291	291	291	291	291	291
Proportion of reported adults	58%	100%	81%	100%	54%	65%	32%	43%	22%
Number of children at farmers market	510	800	800	200	200	2000	2000	500	500
Number of reported children	8	8	8	8	8	8	8	8	8
Estimated number of ill children	14	8	10	8	15	12	25	19	37
Estimated AR (children)	2,7%	1,0%	1,2%	4,0%	7,4%	0,6%	1,2%	3,7%	7,4%
Estimated total diseased	512	299	370	299	555	462	925	694	1387

The total number of visitors (line 1) is varied between 2000 and 5000, the proportion of adult visitors (line 2) is varied between 60% and 90% and the estimated AR among adults (line 4) is varied between 15% and 30% yielding 8 different scenarios; for further comments on calculation steps: see text.

### Environmental investigation

In total, 5 lambs and 5 ewes were displayed on the market, one of them was pregnant and gave birth to twin lambs at 6:30 a.m. on May 4, 2003. Of these, 3 ewes including the one that had lambed tested positive for *C. burnetii*. The animals came from a flock of 67 ewes, of which 66 had given birth between February and June. The majority of the births (57 (86%)) had occurred in February and March, usually inside a stable or on a meadow located away from the town. Six ewes aborted, had stillbirths or abnormally weak lambs. Among all ewes, 17/67 (25%) tested positive for *C. burnetii*.

The percentage of sheep that tested positive in the other 5 sheep flocks in the region ranged from 8% to 24% (8%; 12%; 12%; 16%; 24%).

## Discussion

We have described one of the largest Q fever outbreaks in Germany which, due to its point-source nature, provided the opportunity to assess many epidemiological features of the disease that can be rarely studied otherwise.

In 1954, more than 500 cases of Q fever were, similar to this outbreak, linked to the abortion of an infected cow at a farmers' market[15]. More recently a large outbreak occurred in Jena (Thuringia) in 2005 with 322 reported cases[16] associated with exposure to a herd of sheep kept on a meadow close to the housing area in which the cases occurred.

The first case control study served to confirm the hypothesis of an association between the outbreak and the farmers' market. The fact that only attendance on the second, but not the first day was strongly associated with illness pointed towards the role of the ewe that had given birth in the early morning hours of May 4, 2005. This strong association and the very high attributable fraction among all cases suggested a point source and justified defining cases notified through the reporting system as outbreak cases if they were clinically compatible with Q fever and gave a history of having visited the farmers' market. The point-source nature of the outbreak permitted calculation of the incubation period of cases which averaged 21 days and ranged from 2 to 48 days with an interquartile range of 16 to 24 days. This is compatible with the literature[1]. An additional interview with the two cases with early onset (2 and 4 days after attending the market on May 4, respectively) could not identify any other source of infection. A short incubation period was recently observed in another Q fever outbreak in which the infectious dose was likely very high[17].

The second case control study among persons who visited the market on May 4 demonstrated that both close proximity to the ewe and duration of exposure were important risk factors. This finding was confirmed by the cohort study on vendors which showed that those who worked in a stand close to (within 6 meters) the sheep pen were at significantly higher risk of acquiring Q fever. The study failed to show a significant role of the location of the stand in reference to the wind direction, although we must take into account that the wind was likely not always and exactly as reported by the weather station. However, if the wind had been important at all more cases might have been expected to have occurred among vendors situated at a greater distance to the sheep.

According to statutory surveillance system data, the proportion of clinical cases hospitalized was 25%, similar to the proportion of 21% found in persons pooled from the other studies conducted. Several publications report lower proportions than that found in this investigation: 4% (8/191)[7], 5%[1] and 10% (4/39)[5]), and there was at least one study with a much higher proportion (63% (10/16))[18]. It is unlikely that hospitals reported cases with Q fever more frequently than private physicians because the proportion hospitalized among Q fever patients identified through random telephone calls in the Soest population or those in the two cohorts was similar to that of notified cases. Thus reporting bias is an unlikely explanation for the relatively high proportion of cases hospitalized. Alternative explanations include overly cautious referral practices on the part of attending physicians or the presumably high infectious dose of the organism in this outbreak, e.g. in those cases that spent time in the sheep pen.

The estimated attack rate among adults in the four studies varied between 16% and 33%. The estimate of 23% based on the random sample of persons visiting the market on the second day would seem most immune to recall bias, even if this cannot be entirely ruled out. The estimation based on information about persons accompanying the cases may be subject to an overestimation because these individuals presumably had a higher probability of being close to the sheep pen, similar to the cases. On the other hand the estimate from the cohort study on vendors might be an underestimate, since the vendors obviously had a different purpose for being at the market and may have been less interested in having a look at the sheep. Nevertheless, all estimates were independent from each other and considering the various possible biases, they were remarkably similar. In comparison, in a different outbreak in Germany, in which inhabitants of a village were exposed to a large herd of sheep (n = 1000–2000)[5,7] the attack rate was estimated as 16%. In a similar outbreak in Switzerland several villages were exposed to approximately 900 sheep[19]. In the most severely affected village, the clinical attack rate was 16% (estimated from the data provided)[19]. It is remarkable that in the outbreak described here, the infectious potential of one pregnant ewe – upon lambing – was comparable to that of entire herds, albeit in different settings.

Our estimate of the proportion of serologically confirmed cases that became symptomatic (50% (3/6)) is based on a very small sample, but consistent with the international literature. In the above mentioned Swiss outbreak, 46% of serologically positive patients developed clinical disease[7].

Only approximately half of all symptomatic cases were reported to the statutory surveillance system. Patients who did not seek health care due to mild disease as well as underdiagnosis or underreporting may have contributed to the missing other half. Our estimated 3% attack rate among children is based on a number of successive assumptions and must therefore be interpreted with caution. Nevertheless, sensitivity analysis confirmed that adults had a significantly elevated attack rate compared to children. While it has been suggested that children are at lower risk than adults for developing symptomatic illness[7,8] few data have been published regarding attack rates of children in comparison to adults.

The estimated *C. burnetii *seroprevalence in the sheep flocks in the area varied from 8% to 24%. The 25% seroprevalence in the flock of the exhibited animals together with a positive polymerase chain reaction in an afterbirth in June 2003 suggested a recent infection of the flock[20]. Seroprevalence among sheep flocks related to human outbreaks tend to be substantially higher than those in flocks not related to human outbreaks. The median seroprevalence in a number of relevant studies performed in the context of human outbreaks[7,20,21], was 40% compared to 1% in sheep flocks not linked to human outbreaks[20].

This outbreak shows the dramatic consequences of putting a large number of susceptible individuals in close contact to a single infected ewe that (in such a setting) can turn into a super-spreader upon lambing. There is always a cultural component in the interaction between people and animals, and these may contribute to outbreaks or changing patterns of incidence. During the past decades urbanization of rural areas and changes in animal husbandry have occurred[20], with more recent attempts to put a "deprived" urban population "in touch" with farm animals. Petting zoos, family farm vacations or the display of (farm) animals at a market such as this may lead to new avenues for the transmission of zoonotic infectious agents[20,22-24]. While not all eventualities can be foreseen, it is important to raise awareness in pet and livestock owners as well as to strengthen recommendations where necessary. This outbreak led to the amendment and extension of existing recommendations[25] which now forbid the display of sheep in the latter third of their pregnancy and require regular testing of animals for *C. burnetii *in petting zoos, where there is close contact between humans and animals.

## Conclusion

Due to the size and point source nature this outbreak permitted reassessment of fundamental, but seldom studied epidemiological parameters of Q fever. It also served to revise public health recommendations to account for the changing type and frequency of contact of susceptible humans with potentially infectious animals.

## Abbreviations

AFE = attributable fraction of cases exposed

AR = attack rate

CCS1 = first case control study

CCS2 = second case control study

CDC = Centers for Disease Control and Prevention, Atlanta, USA

CF = complement fixation

ELISA = enzyme-linked immunosorbent assay

IFA = immunofluorescence assay

MA = microagglutination

NCL = national consulting laboratory

OR = odds ratio

RKI = Robert Koch-Institute

RR = relative risk

SARS = severe acute respiratory syndrome

## Competing interests

The author(s) declare that they have no competing interests.

## Authors' contributions

KP headed the investigation, analysed data and wrote the first version of the manuscript. JR and UvT assisted in the design of the study and co-ordinated reporting of cases. AT co-ordinated activities on the local level and conducted exploratory interviews. SB assisted in the design of the study and co-ordinated collection of veterinary data. WH collected veterinary data and assisted with advice on veterinary issues. ML collected data from reporting health departments. PK, SB and CW co-ordinated laboratory confirmatory testing and followed up patients with valvular heart defects and pregnant women. WH assisted with the study design, literature review and writing of the manuscript. UB conceived of the study, analysed data and wrote later versions of the manuscript. All authors read and approved the final manuscript.

## Pre-publication history

The pre-publication history for this paper can be accessed here:


